# Clostridium Tetani Bacteremia From a Suspected Cutaneous Source

**DOI:** 10.7759/cureus.22848

**Published:** 2022-03-04

**Authors:** David Kazadi, Diana Zychowski, Caleb Skipper, Peter Teravskis, Glen T Hansen, Eloy E Ordaya

**Affiliations:** 1 Internal Medicine, University of Minnesota, Minneapolis, USA; 2 Infectious Diseases, University of North Carolina at Chapel Hill, Chapel Hill, USA; 3 Infectious Diseases, University of Minnesota, Minneapolis, USA; 4 Microbiology and Molecular Diagnostics, Hennepin Healthcare, Minneapolis, USA; 5 Infectious Diseases, Mayo Clinic, Rochester, USA

**Keywords:** cellulitis, 16s rrna gene sequencing, bacteremia, tetanus, clostridium tetani

## Abstract

Bacteremia is a rare complication of *Clostridium tetani* infection. To our knowledge, there are only two case reports to date of *C. tetani bacteremia*, both hypothesized to be secondary to a gastrointestinal source. Herein, we report a case of an elderly man with genome sequence-proven *C. tetani* bacteremia from a possible cutaneous source without neuromuscular symptoms.

## Introduction

*Clostridium tetani *is a spore-forming, toxin-producing, pathogenic anaerobic bacteria that typically has a gram-positive staining pattern [1]. *C. tetani* spores are found in soil and the intestinal tract and feces of animals and humans [1, 2]. *C. tetani* can produce tetanolysin and tetanospasmin exotoxins that are responsible for the acute and often fatal neuromuscular disease, tetanus. Tetanospasmin prevents the release of neurotransmitters from inhibitory cells, leading to increased muscle tone and hypersympathetic state [1]. The spores gain entry into the body through a break in the skin barrier and are disseminated through the blood and lymphatics, ultimately leading to toxin production and retrograde axonal transport from the periphery into the CNS, resulting in a reduction in motor nerve inhibition and the pathognomonic muscular spasms [1].

Tetanus can be divided into four different clinical types: generalized, cephalic, localized, and neonatal [3, 4]. Generalized tetanus, the most common manifestation, begins with increased tone or rigidity of facial muscles and leads to descending generalized rigidity [3, 4]. Cephalic tetanus affects the cranial nerve muscles and is almost always associated with otitis media or prior head injury [3, 4]. Localized tetanus is a rare presentation involving persistent muscle contractions in the same anatomic area of the injury or spore inoculation [3, 4]. Clinical manifestations of localized tetanus may be mild and sometimes preceded by the development of generalized tetanus [3, 4]. Neonatal tetanus is a form of generalized tetanus occurring in infants who are born without passive immunity [3, 4].

Unlike the neuromuscular disease, bacteremia represents an under-recognized or rare complication of *C. tetani* infection. Using the MeSH (medical subject headings) terms “Clostridium tetani” and “bacteremia” to search in PubMed yielded only two case reports of patients with *C. tetani *bacteremia, each from a postulated gastrointestinal source [5, 6]. Herein, we describe a case of a patient with proven* C. tetani *bacteremia from a potential skin source.

## Case presentation

An 86-year-old man with a 60-year history of iatrogenic hypothyroidism was brought to the emergency department after being found on the ground for several days following an unwitnessed fall. He could not remember the circumstances of his fall but complained of shortness of breath and left leg pain over the preceding days. He denied having fevers, cough, diarrhea, or any neurological symptoms. His history was also notable for hypertension, obesity, polyarticular gout, hyperlipidemia, and limited mobility. Finally, the patient had known mediastinal lesions concerning for neoplastic disease for which he had declined further evaluation.

**Figure 1:**
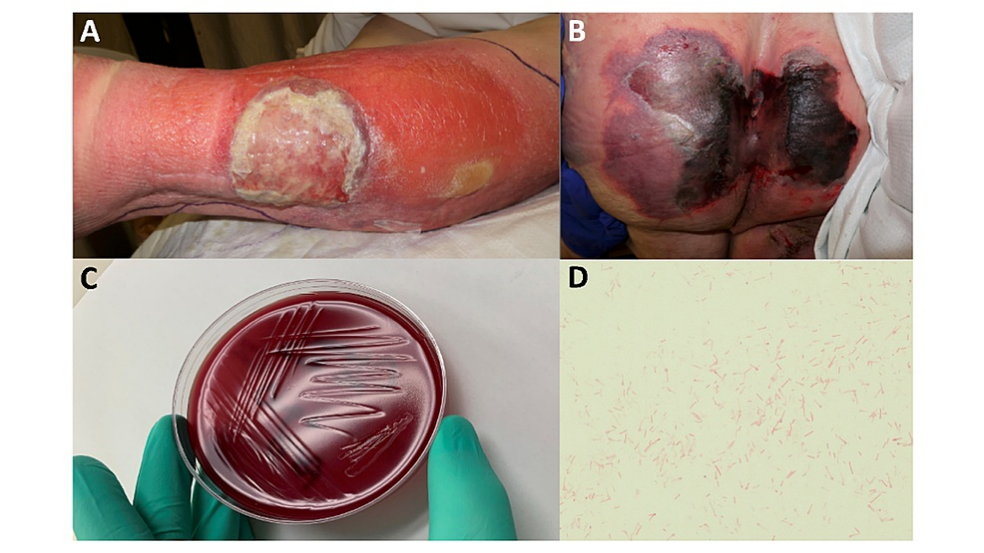
Suspected cutaneous sources (A, B) and identification of Clostridium tetani (C, D). (A) Left lower extremity cellulitis with ulceration. (B) Sacral decubitus ulcer with overlying necrotic eschar. (C) *C. tetani* appearing as swarm without discernible colonies on Brucella Blood agar plate. (D) Gram stain of *C. tetani*. Not unusual for large anaerobic gram-positive rods to decolorize. 100x magnification.

On presentation, he was tachycardic to 120 beats per minute, had a respiratory rate of 20 breaths per minute, but was afebrile and normotensive. Physical exam was remarkable for a blistering ulceration with surrounding erythema in the lateral aspect of his left lower extremity (Figure 1A), and an unstageable sacral ulceration with overlying necrotic eschar (Figure 1B). Laboratory tests were significant for a white blood cell count of 21,000/µL, lactate of 2.3 mmol/L, creatinine of 1.6 mg/dL, procalcitonin of 1.33 ng/mL, C-reactive protein of 377.77 mg/L, a D-dimer of 4662 ng/mL, a creatine kinase of 5914 U/L, a prostate-specific antigen of 167.01 ng/mL, and elevated liver enzymes (Table 1). Computed tomography (CT) angiogram of the chest revealed a left upper lobe segmental pulmonary embolus with right ventricular strain, heterogeneous bilateral ground-glass opacities, and likely progression of neoplastic disease in lymph nodes and bone. Though nonspecific fat stranding was reported around the gallbladder on CT, cholecystitis was ruled out after right upper quadrant ultrasound and nuclear medicine hepatobiliary scan. CT of the abdomen and pelvis also reported colonic ileus and soft tissue thickening around the coccyx without drainable fluid collection, but with several foci of superficial gas.

**Table 1:** Laboratory tests on admission and at discharge Values of laboratory tests on admission and around discharge. For some tests with variable results, a range of values is reported. Discharge occurred about five weeks after admission, and discharge tests were generally completed five-to-seven weeks after admission, except for creatine kinase (four days after admission). Some tests were not repeated after admission.

Laboratory test (units; reference range)	Admission	Discharge
Sodium (mmol/L; 136-145)	134	131-137
Potassium (mmol/L; 3.5-5.1)	3.5	25
Chloride (mmol/L; 98-107)	97	102
Bicarbonate (mmol/L; 22-29)	25	25
Urea nitrogen (mg/dL; 8-26)	42	18-30
Creatinine (mg/dL; 0.7-1.2)	1.6	1
Glucose (mg/dL; 74-100)	119	88
Aspartate aminotransferase (U/L; ≤34)	162	14
Alanine aminotransferase (U/L; ≤55)	64	8
Alkaline phosphatase (U/L; 40-150)	155	108
Total bilirubin (mg/dL; 0.2-1.2)	1.8	0.4
Direct bilirubin (mg/dL; ≤0.5)	1.3	-
Thyroid-stimulating hormone (µIU/mL; 0.35-4.94)	5.04	3.37
Free thyroxine (ng/dL; 0.70-1.48)	0.4	-
B-type natriuretic peptide (pg/mL; ≤99)	67	-
Cardiac Troponin I (ng/mL; ≤0.028)	≤0.028 x2	-
D-dimer (FEU ng/mL; 0-500)	4662	-
Creatine kinase (U/L; 39-308)	5914	420
White blood cell count (K/cmm; 4-11)	21	10.28
Hemoglobin (g/dL; 13.5-17.9)	12.2	7.7
Hematocrit (%; 41-54)	37.3	24.5
Platelet count (K/cmm; 150-400)	224	380
Lactic acid (mmol/L; 0.5-2.2)	1.3	-
C-reactive protein (mg/L; ≤5)	377.77	63.50
Procalcitonin (ng/mL; ≤0.09)	1.33	0.19
Prostate-specific antigen (ng/mL; ≤3.99)	167.01	-

The patient received intravenous fluids and anticoagulation with therapeutic heparin infusion (ultimately transitioned to apixaban). After samples for blood culture were collected, antibiotic therapy with piperacillin-tazobactam 3.375 gm every six hours and vancomycin 2000 mg daily was initiated, and the patient was admitted to the hospital for further management.

Anaerobic blood cultures tested positive 24 hours after collection with large gram-positive rods visualized under microscopy (Figures 1C-1D) and identified as “non-perfringens *Clostridium spp*.” Repeat anaerobic cultures from the following day also grew non-perfringens gram-positive rods. Positive cultures were preliminarily identified as *Clostridium tetani* by mass spectrometry. *Clostridium tetani* isolation was further confirmed by 16S ribosomal ribonucleic acid (rRNA) gene sequencing (GenBank Accession Number OK598000). Immunization history was reviewed and revealed that the patient had received the tetanus, diphtheria, and pertussis combination vaccine (TDaP) seven years prior to this admission. Because there were no signs of neuromuscular dysfunction despite bacteremia, tetanus immune globulin was not administered. He underwent debridement of his sacral ulcer, where polymicrobial growth - including isolation of *Clostridium inocuum* and *Clostridium sporogenes* - was noted. No culture was obtained from the patient’s left lower extremity cellulitis given the lack of purulence. Over the following days, his leg and sacral wounds improved, his kidney function and white blood cell count normalized (Table 1), and surveillance blood cultures resulted negative. His antibiotic regimen was ultimately narrowed to oral amoxicillin 1 g three times daily to complete four weeks of total antibiotic treatment, and he was discharged to a rehabilitation unit for continued convalescence after five weeks in the hospital.

## Discussion

We describe a case of a patient with *Clostridium tetani *bacteremia from a suspected skin and soft tissue source. To our knowledge, this is only the second such reported case of *C. tetani* bacteremia in the United States and the first with a probable skin source. Hallit et al. described the case of a woman with generalized weakness, diarrhea, and abdominal pain, who was found to have *C. tetani* bacteremia thought to have originated from a ventral hernia [5]. Lai et al. reported the case of a cirrhotic man from Taiwan who presented with general weakness, fever, and vomiting and was found to have *C. tetani *bacteremia, a putative complication from transarterial embolization for the management of hepatocellular carcinoma [6]. In both cases, the patients presented with gastrointestinal complaints without tetanus symptoms, and they both subsequently improved on oral metronidazole.

Our patient's breach in skin integrity combined with prolonged contact with soil makes skin and soft tissue the likeliest route of exposure to *C. tetani* spores. It is also possible that the proximity of his sacral ulcer to the anal opening heightened the risk of bacterial spread from the gastrointestinal tract to the decubitus ulcer, whence it then could have hematogenously or lymphatically spread. Cultures from the sacral ulcer debridement showed polymicrobial growth with isolation of *Clostridium innocuum* and *Clostridium sporogenes* but not *C. tetani*. This may reflect an accurate sampling of cultured isolates or could be reflective of partial elimination of *C. tetani* following antibiotic treatment. A gastrointestinal source is certainly plausible, though bacterial translocation followed by bacteremia would typically be expected with breach to the gastrointestinal epithelium, as may be seen with perforation or gastrointestinal inflammation [7]. Despite ileus being noted on imaging, our patient did not have evidence of intestinal obstruction, colitis, or other signs or symptoms concerning for a gastrointestinal infectious process.

Similar to the case described by Hallit et al., our patient, fortunately, had up-to-date immunizations, including having received the tetanus toxoid-containing booster seven years before his presentation to the emergency department. He did not exhibit neuromuscular dysfunction, so we hypothesize that adequate immunization likely protected him against developing generalized or cephalic tetanus, though mild localized tetanus arguably remains possible. However, despite this protection, the patient developed severe sepsis, underlining the putative non-toxin-mediated pathogenicity of *C. tetani.* Another possibility is that the *C. tetani* strain produced defective or no toxins, highlighting a presumed non-toxin-dependent pathogenicity.

The diagnosis of tetanus is usually made clinically and does not routinely require isolation of *C. tetani* [3, 4]. Laboratory identification of *C. tetani* has traditionally been challenging and at times inconclusive, relying on various techniques, including culture in pre-reduced anaerobically sterilized (PRAS) media, analytical profile index methods, and toxin production in mice [3, 8-11]. More recently, and as our case illustrates, identification of *C. tetani* has leveraged confirmatory testing modalities (including matrix-assisted laser desorption/ionization-time of flight (MALDI-TOF) mass spectrometry and 16S rRNA genome sequencing) that arguably may be less labor-intensive and more accurate than traditional methods [8, 12]. The increasing utilization of such tools for identifying clostridial species suggests that perhaps *C. tetani* bacteremia may become more recognized in the future.

## Conclusions

This report describes a rarely recognized presentation in which *C. tetani* can cause invasive disease such as bacteremia without neuromuscular dysfunction and also underscores the importance of up-to-date immunizations and the utility of traditional and novel microbiological identification tools for clostridial infections.
